# Bacterial extracellular vesicles in the microbiome of first-pass meconium in newborn infants

**DOI:** 10.1038/s41390-022-02242-1

**Published:** 2022-08-09

**Authors:** Jenni Turunen, Mysore V. Tejesvi, Marko Suokas, Nikke Virtanen, Niko Paalanne, Anna Kaisanlahti, Justus Reunanen, Terhi Tapiainen

**Affiliations:** 1 Research Unit of Clinical Medicine, University of Oulu, Oulu, Finland; 2 Biocenter Oulu, University of Oulu, Oulu, Finland; 3 Ecology and Genetics, Faculty of Science, University of Oulu, Oulu, Finland; 4 Research Unit of Translational Medicine, University of Oulu, Oulu, Finland; 5 Department of Pediatrics and Adolescent Medicine, Oulu University Hospital, Oulu, Finland

## Abstract

**Background:**

Bacterial extracellular vesicles (EVs) are more likely to cross biological barriers than whole-cell bacteria. We previously observed EV-sized particles by electron microscopy in the first-pass meconium of newborn infants. We hypothesized that EVs may be of bacterial origin and represent a novel entity in the human microbiome during fetal and perinatal periods.

**Methods:**

We extracted EVs from first-pass meconium samples of 17 newborn infants and performed bacterial 16S rRNA gene sequencing of the vesicles. We compared the EV content from the meconium samples of infants based on the delivery mode, and in vaginal delivery samples, based on the usage of intrapartum antibiotics.

**Results:**

We found bacterial EVs in all first-pass meconium samples. All EV samples had bacterial RNA. Most of the phyla present in the samples were Firmicutes (62%), Actinobacteriota (18%), Proteobacteria (10%), and Bacteroidota (7.3%). The most abundant genera were *Streptococcus* (21%) and *Staphylococcus* (17%). The differences between the delivery mode and exposure to antibiotics were not statistically significant.

**Conclusions:**

Bacterial EVs were present in the first-pass meconium of newborn infants. Bacterial EVs may represent an important novel feature of the gut microbiome during fetal and perinatal periods.

**Impact:**

- We show that bacterial extracellular vesicles are present in the microbiome of first-pass meconium in newborn infants.
- This is a novel finding. To our knowledge, this is the first study to report the presence of bacterial extracellular vesicles in the gut microbiome during fetal and perinatal periods.
- This finding is important because bacterial extracellular vesicles are more likely to cross biological barriers than whole-cell bacteria. Thus, the early gut microbiome may potentially interact with the host through bacterial EVs.

## Introduction

The first stool after birth, i.e., first-pass meconium, has previously been proposed to be a possible proxy of fetal gut microbiome^1,2^ because meconium may be formed in utero before birth, and many recent studies using 16S rRNA gene sequencing have identified a unique microbiota in meconium.^2–6^ The observed differences in meconium microbiota between neonates born by vaginal delivery and caesarean section (C-section), however, suggest that the development of the gut microbiota may mostly be a perinatal event.^6–8^ Furthermore, a recent study by Kennedy et al. found that meconium may not inhabit bacteria until later after birth.^9^ Finally, some earlier microbiome findings in the first stool may be explained by contamination rather than true contact with microbial nucleic acids in utero.^9,10^

Even though true colonization by whole-cell bacteria appears to be unlikely in the fetal period, according to the present evidence, it is hypothetically possible that the fetus may be exposed to microbial DNA in utero in alternative ways. The role of extracellular vesicles (EVs) in the human gut microbiome is an understudied topic. EVs are cell-derived small particles known to carry various molecules, including RNA and DNA, and they can cross biological barriers.^11^ The functions of EVs are thought to include cell-to-cell communications and signaling with cargo contained within.^11^ It has been found that both gram-negative^12–15^ and gram-positive^14–17^ bacteria can secrete EVs in the form of outer membrane vesicles and membrane vesicles, respectively. These particles can have many different roles, often related to bacterial survival, depending on the species that produce them.^12^ Recently, interest in bacterial EVs, or bEVs, has increased in the medical field, and their role and usage as biomarkers in human health have been explored in metabolic diseases^18^ and cancers, such as gastrointestinal,^19^ gastric,^20^ and colorectal cancer.^21^ The role of bacterial EVs in the early human microbiome might be biologically important since they are more likely to cross biological barriers than whole-cell bacteria.^11^ In our previous study of the first-pass meconium microbiome, we found EV-sized particles in meconium samples using electron microscopy and nanoparticle tracking analysis.^6^

We hypothesized that the previously observed EVs in first-pass meconium^6^ may be of bacterial origin and represent a novel feature of the human microbiome in fetal and perinatal periods. Here, we set out to extract EVs from first-pass meconium samples of newborn infants and characterize bacterial RNA from them using 16S rRNA gene sequencing. Furthermore, we compared the findings between infants born by C-section, vaginal delivery, and vaginal delivery with intrapartum antibiotic exposure during birth.

## Patients and methods

### Study design and study population

The research plan was reviewed and approved by the Ethical Committee of the Northern Ostrobothnia Hospital District at Oulu University Hospital, Finland (decision number EETTMK:3/2016). The families gave their written informed consent prior to sample collection. All relevant guidelines and regulations regarding clinical research were followed during the study. Meconium samples were collected from 17 term newborn infants at Oulu University Hospital, Finland. Five infants were born by C-section, six infants were born by vaginal delivery without intrapartum antibiotics, and six were born by vaginal delivery with intrapartum antibiotic exposure.

### Sample collection

First-pass meconium samples were collected from a diaper within 24 h of birth. This collection method was chosen based on its non-invasiveness and due to a previous meconium study by us, where we found no bacteria from diapers themselves.^2^ The collection was performed by either a midwife in the delivery room or by families or a study nurse in the ward. Samples were immediately stored at −20 °C, and later at −80 °C before further processing.

### Extracellular vesicle extraction

Meconium samples (100–900 mg) were transferred to 50 ml Falcon tubes with 15 ml of sterile-filtered phosphate-buffered saline (PBS). Samples were disrupted and resuspended by pipetting, using a 25 ml pipet, then centrifuged at 4 °C, 14,000 g for 30 min. Supernatants were moved to new tubes and centrifuged again using the same settings. The resulting supernatants were filtered into new tubes through a 40 µm nylon filter (Falcon), then filtered again using a 0.45 µm PES filter (Biofil).

Samples were concentrated with Amicon® Ultra-15 Centrifugal filter units (100 kDa). The units were washed before use by adding 10 ml of PBS and centrifuging at 3000 g for 5 min before removing the PBS. Samples were added to the tubes, centrifuged at 4 °C, 3000 g for 30 min, and the concentrates were collected.

EVs were isolated from the samples using Exo-Spin™ Mini-Columns (Cell Guidance Systems, Cambridge, England). The columns were prepared by removing caps and plugs and spinning down at 50 g for 10 s, then equilibrated by adding 200 µl of PBS and spinning again at 50 g for 10 s. Then, 200 μl of concentrated sample was added to the columns and centrifuged at 50 g for 1 min. Finally, the columns were moved to collection tubes, 200 µl of PBS was added, and the columns were centrifuged at 50 g for 1 min to eluate the vesicles.

Vesicle samples were purified using gradient ultracentrifugation. Gradient solutions were prepared with Optiprep solution (Fisher Scientific) and pipetted in 2.5 ml layers, starting with a layer of 40% solution on the bottom, followed by layers of 20%, 10%, and 5% solutions (10 ml in total per tube). Then, 200 µl of vesicle sample was added on top of the gradient and centrifuged at 4 °C, 100,000 g for 18 h. The resulting ten fractions of 1 ml each were collected and labeled with numbers starting at the top of the tube, e.g., the first 1 ml to be collected was fraction 1 and the last was fraction 10. Fractions 5–8 would be used in further experiments and were washed to remove any remaining impurities. Then, 9 ml of PBS was added to each of these fractions, mixed, and centrifuged at 4 °C, 100,000 g for 2.5 h. The resulting pellets were resuspended in 100 μl of PBS, and samples were stored in parafilm sealed tubes at 4 °C, awaiting further procedures.

### Vesicle RNA extraction, cDNA conversion, and PCR amplification

RNA was extracted using a modified protocol from the exoRNeasy Plasma Midi Kit (Qiagen). For each sample, 25 µl of four vesicle fractions were combined in a 1.5 ml microcentrifuge tube, followed by the addition of 500 µl of Qiazol reagent. Control samples were extracted from a nuclease-free tube without any samples. All samples were mixed and incubated at room temperature for 5 min. After incubation, 78 µl of chloroform was added to the samples and mixed by inverting the tubes several times. Samples were incubated again for 3 min at room temperature, and phases were separated by centrifugation at 12,000 g for 15 min at 4 °C. The upper aqueous phase was transferred to a clean tube and mixed with 2 volumes of ethanol. Column purification steps were performed according to the manufacturer’s instructions, and RNA concentration was measured from eluate using a Nanodrop spectrophotometer (Thermo Scientific). Two empty, sterile tubes were included in the RNA extraction as negative controls alongside the vesicle samples. According to the kit guidelines, we performed polymerase chain reactions (PCR) on the RNA samples to ensure that the samples contained no DNA. Samples were processed alongside one negative and one positive control. We performed agarose gel electrophoresis to see whether DNA fragments have been produced during PCR, and we found no positive signal from RNA or negative control samples.

RNA was converted to cDNA with an iScript cDNA synthesis kit (Bio-Rad) following the manufacturer’s instructions. Then, 2 µl (approximately 20 ng according to Nanodrop) of RNA and 2 µl (final concentration 0.5 µM) of 16S rRNA specific primer 515F (5′-GTGCCAGCMGCCGCGGTAA-3′) as well as 1x iScript reaction buffer were used in a total volume of 20 µl. Three empty tubes were included in the RT-PCR process as negative controls.

For amplification, the V4-V5 variable region of the 16S small ribosomal unit gene was amplified with primers 519F (5′-CAGCMGCCCGCGGTAATWC-3′) and 926R (5′-CCGTCAATTCCTTTRAGTTT-3′). The 519F primer contained at the beginning an additional Ion Torrent sequencing 30 bp long adapter sequence, a 9 bp long unique barcode sequence for each sample, and a single nucleotide linker A. The 926R primer contained an Ion Torrent adapter sequence trP1 in the beginning. PCR were performed in 30 µl volumes containing 1x Phusion Flash High-Fidelity Master Mix (Thermo Fisher Scientific), 0.75 µM forward and reverse primers, and 5 µl of converted cDNA. After an initial 3-min denaturation at 98 °C, the following conditions were used for 30 cycles: 98 °C for 10 s, 64 °C for 10 s, and 72 °C for 30 s. The final extension step was carried out at 72 °C for 5 min. The success of PCR was confirmed with an Agilent Bioanalyzer.

### Amplicon sample processing and sequencing

PCR reactions were cleaned with the Agencourt AMPure XP PCR purification system (Beckman Coulter) and quantified with an Agilent Bioanalyzer using a DNA 1000 analysis kit (Agilent). Thereafter, samples were pooled at equimolar ratios, and the pool was further purified with Ampure XP, checked for purity with a bioanalyzer, and the concentration was quantified with Quant-iT PicoGreen assay. Sequencing was performed at the Biocenter Oulu Sequencing Center (Oulu, Finland) with an Ion Torrent PGM sequencer. The sequencing was performed with an Ion PGM Hi-Q View template kit using a 400 bp templating program, an Ion PGM Hi-Q View Sequencing kit with 850 cycles, and a 318 v2 chip.

### Analysis

Analysis was performed using Quantitative Insights Into Microbial Ecology 2, also known as QIIME2 (version 2021.2),^22^ and reads shorter than 200 bp were filtered out before further preprocessing. Reads were demultiplexed, and QIIME2-implemented DADA2^23^ was used to denoise reads and filter out chimeric reads. Reads were trimmed at base 15 and truncated at base 250 based on the quality plots created by QIIME2 in the demultiplexing step. We used negative control samples prepared during the RNA extraction and RT-PCR and an R package decontam (version 1.8.0) to filter out contaminant reads from the vesicle samples with the prevalence-based method and a threshold of 0.5.^24^ For taxonomic analysis, taxa identified as mitochondria Eukaryota, Cyanobacteria, and Archaea were removed from the data. After preprocessing, 597,878 reads remained for analysis. For alpha and beta diversity analyses, reads were rarefied at a sampling depth of 22,742.

We used the Shannon index and observed features as metrics for within-sample diversity, known as alpha diversity, and the Kruskal–Wallis *H* was used as a statistical test for the alpha diversity results. For between-sample diversity, known as beta diversity, we analyzed the samples with principal coordinate analysis using Bray–Curtis dissimilarity, Jaccard Index, Unweighted UniFrac, and Weighted UniFrac as our metrics. These results were statistically tested using PERMANOVA analysis. Alpha and beta diversities were visualized with RStudio (version 2021.9.2.382 with R version 4.1.2) and packages ggplot2 (version 3.3.5), grid (4.0.3), and gridExtra (2.3). The figures were finalized using Inkscape (version 1.1) All statistical tests used the *p* value as a measure of significance. A value of 0.05 or less was considered statistically significant. Taxonomic analysis was performed with the Silva database (version 138)^25^ using a self-trained classifier. Differential abundance between different sample types was analyzed using QIIME2-implemented analysis of composition of microbiomes (ANCOM).^26^ Taxonomy was visualized with Krona.^27^ The raw sequences were submitted to Genbank under the bioproject accession number PRJNA816091.

We used QIIME2-implemented Phylogenetic Investigation of Communities by Reconstruction of Unobserved States (PICRUSt2)^28^ to investigate functions, or metabolic pathways, involved with the bacterial taxa found in the samples. The differential abundance between different sample types was analyzed using ANCOM and ALDEx2.^29^

## Results

Altogether, 17 first-pass meconium samples were processed and analyzed. Twelve samples were obtained from infants born by vaginal deliveries and five from those born by C-section. Of the 12 infants born by vaginal delivery, six had been exposed to antibiotic treatment during birth. All infants born by C-section were exposed to antibiotics during birth (Table 1).

**Table 1:** Characteristics of the newborn infants.

Population characteristics	Vaginal delivery (*n* = 12)	Caesarean section (*n* = 5)	All (*n* = 17)
*Maternal characteristics*			
Maternal age, mean (SD)	28.9 (4.9)	27.3 (3.1)	28.4 (4.4)
Number of siblings mean (SD)	2.0 (2.6)	1.4 (1.1)	1.8 (2.2)
Maternal asthma, *N* (%)	0	0	0
Maternal allergy, *N* (%)	3 (25)	1 (20)	4 (24)
GDM	0	1 (20)	1 (6)
Smoking during pregnancy	0	0	0
Group B streptococcus positive^a^	6 (50)	1 (20)^b^	7 (41)
Antibiotics during pregnancy^b^	0	3 (60)	3 (18)
Antibiotics during delivery^c^	6 (50)	5 (100)	11 (65)
*Newborn characteristics*			
Gestational age (weeks) mean (SD)	39.1 (1.1)	38.6 (2.1)	39 (1.4)
Birth weight (g) mean (SD)	3450 (550)	3620 (950)	3500 (660)
Apgar 1 min mean (SD)	8.4 (1.4)	9.2 (0.8)	8.7 (1.2)
Apgar 5 min mean (SD)	9.0 (0.7)	9.6 (0.5)	9.2 (0.7)
Apgar 15 min mean (SD)	9.3 (0.6)	9.8 (0.4)	9.4 (0.6)
Perinatal antibiotics^d^	0	1 (20)	1 (5.9)

*GDM* gestational diabetes mellitus.

^a^*Str. agalactiae* screening was not performed in two cases.

^b^In the caesarean section group, one mother had received pivmecillinam. The antibiotic used was not recorded for two cases in the section group.

^c^In the vaginal delivery group, five mothers received benzyl penicillin, and one mother received clindamycin. In the caesarean section group, four mothers received cefuroxime, and one mother received clindamycin.

^d^One child in the caesarean section group received intravenous benzyl penicillin.

### Microbial composition of extracellular vesicle samples

**Fig. 1:**
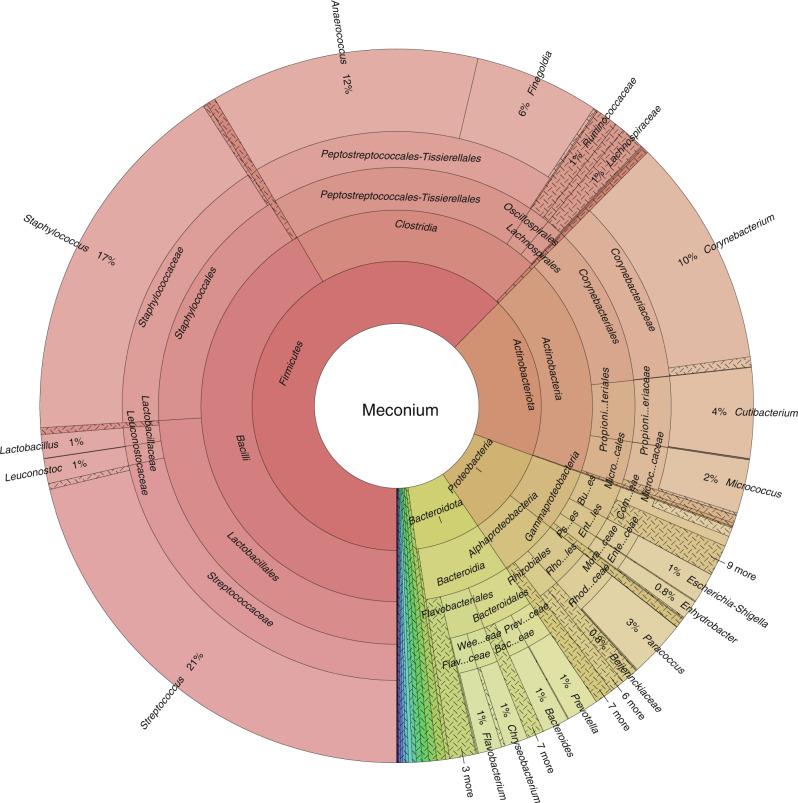
Taxonomic composition of bacterial EVs in the first-pass meconium of newborn infants. Taxonomic levels are presented from the phylum level (innermost circle) to the genus level (outermost circle). The figure was generated using the Krona program.

All EV samples had bacterial RNA. Most of the phyla present in the samples were Firmicutes (62%), Actinobacteriota (18%), Proteobacteria (10%), and Bacteroidota (7.3%) (Fig. 1 and Supplementary information 1). The most abundant genera were *Streptococcus* (21%), *Staphylococcus* (17%), *Anaerococcus* (12%), and *Corynebacterium* (10%) (Fig. 1 and Supplementary information 1).

### Impact of delivery mode and intrapartum antibiotics on microbial findings of EVs

**Fig. 2:**
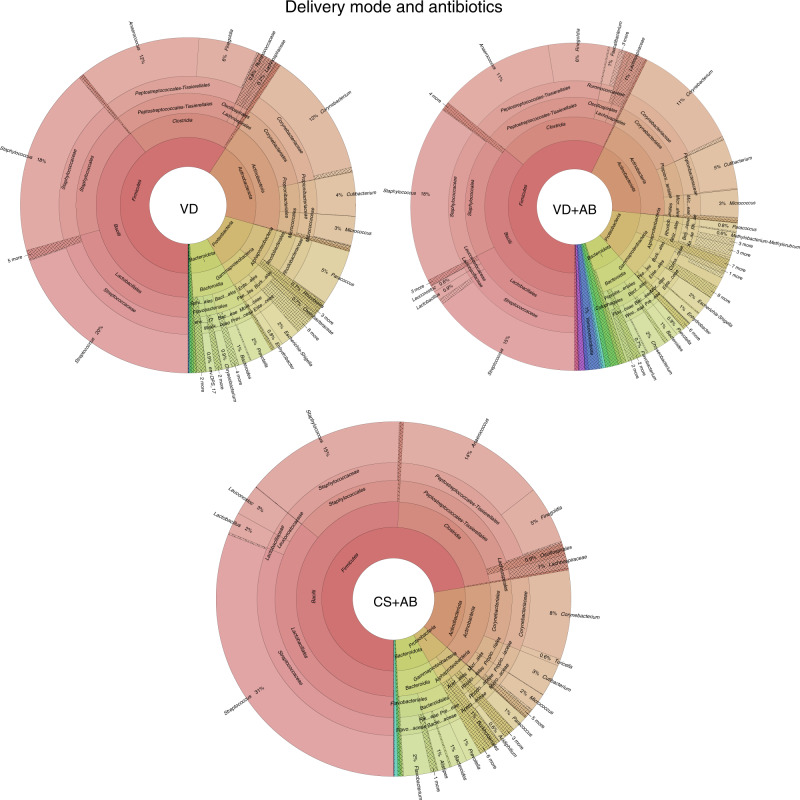
Taxonomic composition of bacterial EVs in the first-pass meconium of newborn infants according to perinatal events. Taxonomic levels are presented from the phylum level (innermost circle) to the genus level (outermost circle). The figure was generated using the Krona program. VD indicates infants born by vaginal delivery, VD+AB indicates those born by vaginal delivery and exposed to antibiotics during delivery, and CD+AB indicates those born by caesarean section and exposed to antibiotics during delivery.

We made comparisons between the taxonomic compositions of vaginal delivery samples (VD), vaginal delivery samples with intrapartum antibiotics (VD+AB), and C-section samples with intrapartum antibiotics (CS+AB). All sample types consisted mostly of phyla Firmicutes (VD: 59%, VD+AB: 57%, CS+AB: 72%), Actinobacteriota (VD: 20%, VD+AB: 19%, CS+AB: 14%), Proteobacteria (VD: 12%, VD+AB: 12%, CS+AB: 5.2%), and Bacteroidota (VD: 7.5%, VD+AB: 6.9%, CS+AB: 7.4%) (Fig. 2 and Supplementary information 2). The most abundant genera in all groups were Streptococcus (VD: 19%, VD+AB: 15%, CS+AB: 31%) and Staphylococcus (VD: 18%, VD+AB: 18%, CS+AB: 15%) (Fig. 2 and Supplementary information 2). The VD+AB group had a slightly higher abundance of low-frequency taxa than the VD and CS+AB.

We performed ANCOM analysis to find differentially abundant taxa between the delivery mode and antibiotics groups. We found Patescibacteria to be differentially abundant on the phylum level, but no differentially abundant genera were found.

**Fig. 3:**
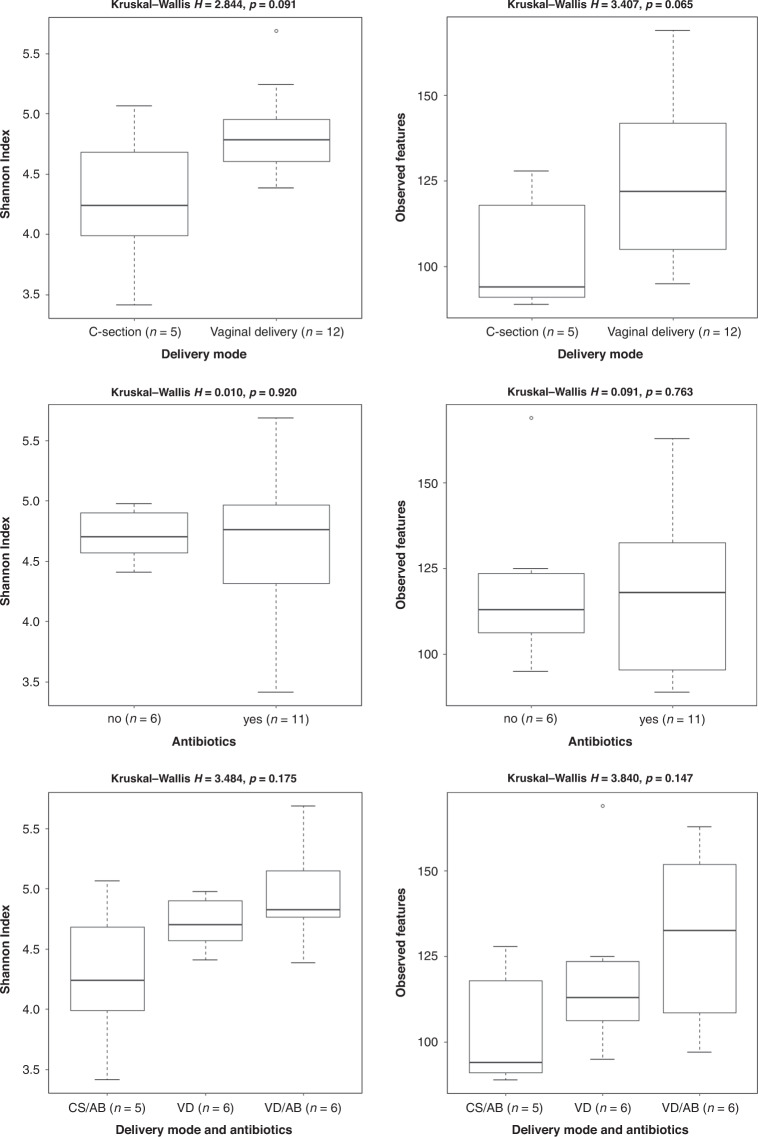
Alpha diversity of bacterial extracellular vesicles in the first-pass meconium based on delivery mode, antibiotics, and delivery mode and antibiotics combined. On the *x*-axis: study subject groups and group sizes. On the *y*-axis: metric used (Shannon index and observed features) and the value of the diversity metric. On top is the statistical significance test Kruskal–Wallis *H* alongside the *p* value of the test.

The within-sample diversity of samples and the number of observed features were lower in samples from infants born by C-section than in those born by the vaginal route, but the differences were not statistically significant (Shannon index *p* = 0.091 and observed features *p* = 0.065) (Fig. 3). There were no differences in the biodiversity of samples in infants born by vaginal delivery according to intrapartum antibiotic exposure (Shannon index *p* = 0.920 and observed features *p* = 0.763) (Fig. 3).

**Fig. 4:**
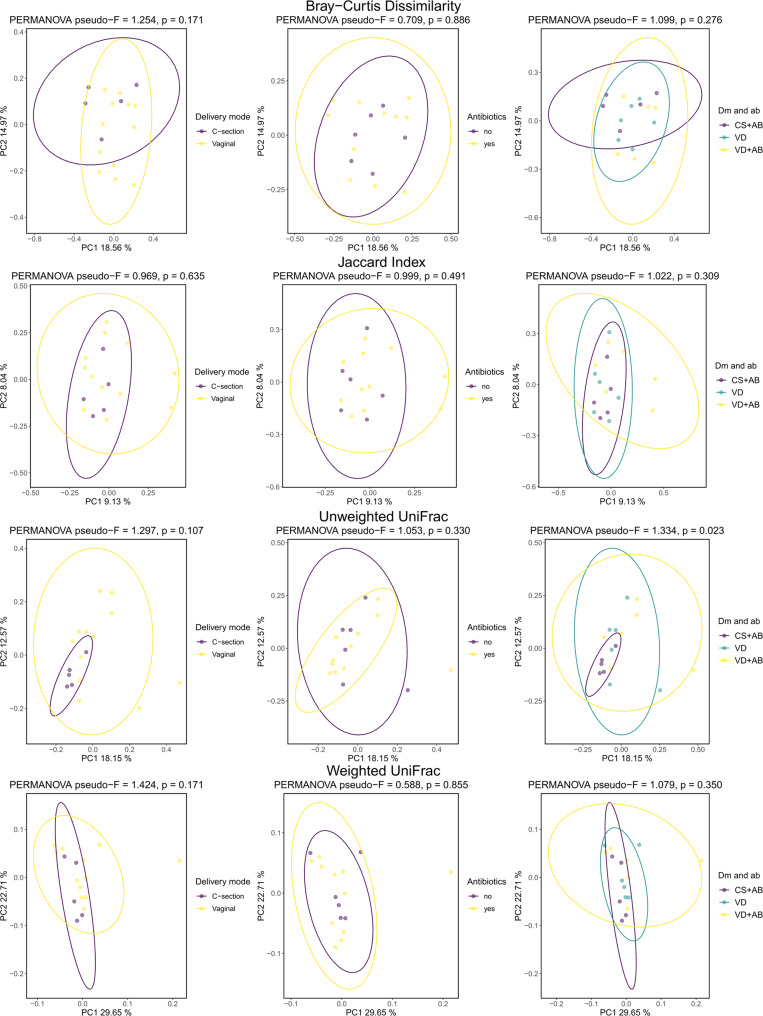
Beta diversity of samples based on delivery mode, antibiotics, and the combination of the two. Beta diversity metrics used in order from top to bottom: Bray–Curtis Dissimilarity, Jaccard Index, unweighted UniFrac, and weighted UniFrac. PERMANOVA was used as a statistical test. In delivery mode and antibiotics (dm and ab), variable CS+AB: C-section and intrapartum antibiotics, VD: vaginal delivery without intrapartum antibiotics, VD+AB: vaginal delivery and intrapartum antibiotics.

Regarding beta diversity, there was no obvious clustering of meconium samples according to the delivery mode or exposure to antibiotics. None of the metrics showed significant differences when comparing delivery mode or the usage of intrapartum antibiotics (Fig. 4). When the delivery mode and the usage of intrapartum antibiotics were combined, a significant difference was found in Unweighted UniFrac (0.023) (Fig. 4). Otherwise, no significant differences were found (Fig. 4).

### PICRUSt2 analysis of the metabolic pathways involved with bEV-secreting bacteria

**Fig. 5:**
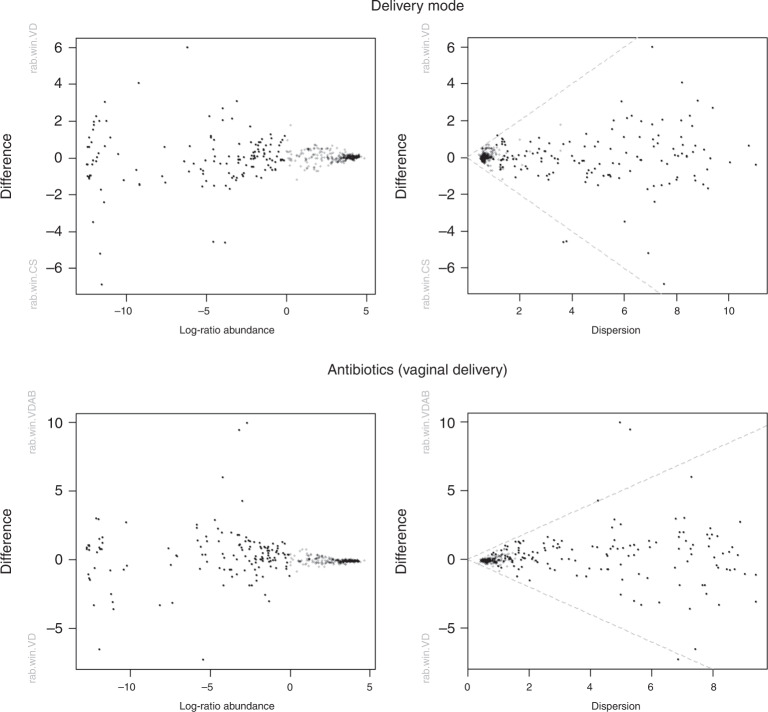
Differential abundance analysis of predicted metabolic pathways using ALDEx2 in Rstudio. On top, the analysis based on the delivery mode, and at the bottom, the analysis based on the intrapartum antibiotic usage in vaginal delivery samples. The left side’s MA plots depict the relationship between relative abundance and difference in pathways. The right side’s effect plots depict the relationship between difference and dispersion in pathways. Statistically significant features would be shown as red dots. On the *x*-axis in the MA plots are the clr values for the features. Welch’s *t*-test was performed as a statistical test, and results with *p* values of less than 0.05 were considered statistically significant.

PICRUSt2 analysis identified 402 metabolic pathways across all meconium samples, which were further explored in metacyc.org. The 20 most frequent pathways found in the samples are listed in Table 2. These predicted metabolic pathways are involved in utilizing glucose and other sugars for energy metabolism, biosynthesis of nucleotides and amino acids, and membrane structure and protein biosynthesis. We performed ALDEx2 analysis to calculate differential abundance in the delivery mode and antibiotic groups, but no significant differences were found (Fig. 5). We also performed ANCOM analysis to confirm the results, and no significant differences were found again.

**Table 2:** The most common predicted metabolic pathways produced by PICRUSt2 in meconium samples by relative frequency.

Metabolic pathway	Pathway synonym	Frequency (%)
PWY-3781	Aerobic respiration I (cytochrome c)	1.010
PWY-7111	Pyruvate fermentation to isobutanol (engineered)^a^	0.927
PWY0-1319	CDP-diacylglycerol biosynthesis II	0.765
PWY-5667	CDP-diacylglycerol biosynthesis I	0.765
ANAGLYCOLYSIS-PWY	Glycolysis III (from glucose)	0.748
PWY-5101	L-isoleucine biosynthesis II	0.748
PHOSLIPSYN-PWY	Superpathway of phospholipid biosynthesis I (bacteria)	0.748
PWY-5484	Glycolysis II (from fructose 6-phosphate)	0.743
NONOXIPENT-PWY	Pentose phosphate pathway (non-oxidative branch) I	0.737
GLYCOLYSIS	Glycolysis	0.736
PWY-7221	Guanosine ribonucleotides de novo biosynthesis	0.732
PWY4FS-7	Phosphatidylglycerol biosynthesis I (plastidic)	0.729
PWY4FS-8	Phosphatidylglycerol biosynthesis II (non-plastidic)	0.729
ANAEROFRUCAT-PWY	Homolactic fermentation	0.727
CALVIN-PWY	Calvin–Benson–Bassham cycle	0.726
PWY-6121	5-Aminoimidazole ribonucleotide biosynthesis I	0.715
PWY-6122	5-Aminoimidazole ribonucleotide biosynthesis II	0.712
PWY-6277	Superpathway of 5-aminoimidazole ribonucleotide biosynthesis	0.712
PWY-5686	UMP biosynthesis I	0.702
PWY-7229	Superpathway of adenosine nucleotides de novo biosynthesis I	0.700

^a^This is an engineered pathway. It does not occur naturally in any known organism and has been constructed in a living cell by metabolic engineering.

## Discussion

In the present study, we showed that the microbiome of first-pass meconium contains bacterial EVs. As bacterial EVs are likely able to cross biological barriers,^11^ our findings indicate that the biological impact of bacterial EVs on the host should be explored further in the microbiome research of fetal and perinatal periods.

Sterile conditions are often defined as the complete absence of living micro-organisms. According to the present evidence, it may be likely that the fetus is sterile in the sense that there are few or no living bacteria present in the fetal gut or other organs.^9,10^ Previous studies have mainly concentrated on bacterial DNA of the meconium microbiome,^2–4,7,8,30^ leaving fungi, viruses, and protozoa, as well as other particles containing microbial nucleic acids largely unexplored. However, our study shows that bacterial EVs are already present in the first-pass meconium. Whether these microbiome-derived particles are immunologically or in other ways interacting with the host should be investigated.

The origin of bacterial EVs in the meconium was not investigated in this study, though based on the genera identified in the samples, we can speculate. Logically, there are two possibilities: either meconium contains live whole-cell bacteria that secrete vesicles, or these vesicles have appeared in meconium already in utero from the maternal microbiome through placenta or amniotic fluid. In addition, the presence of bEVs could be explained by the combination of these routes.

The taxonomy of our bEV samples aligns with earlier findings of bacterial DNA in the meconium. We found Firmicutes to be the dominant phylum in our bEV samples, which is a common gut phylum in humans. At the genus level, the most abundant taxa were *Streptococcus* and *Staphylococcus*, alongside taxa such as *Anaerococcus*, *Escherichia-Shigella*, *Prevotella*, and *Lactobacillus* (Supplementary information 1), all of which are known colonizers of the gut and other parts of the digestive system. In our previous work, we characterized the meconium microbiome and obtained similar results using the same methods.^6^ This indicates that the bEVs we found most likely were secreted by live bacteria present in meconium during the early colonization process. In PICRUSt2 analysis, we found metabolic pathways mostly indicating processes related to the viability of bacteria, as the most common pathways were related to energy metabolism and biosynthesis of membrane structures, nucleotides, and amino acids. We also found taxa, such as *Cutibacterium*, *Corynebacterium*, and *Micrococcus*, which are commensal bacteria on the skin. The findings of skin bacteria in the meconium samples are understandable because the first-pass meconium samples were collected from diapers. While the diaper collection method may have inflated the abundance of skin commensals in our samples, we believe that the diapers themselves have not contributed to the bacterial composition. In a previous study, we analyzed diapers and found no positive bacterial signal.^2^

When comparing samples based on the delivery mode in the present study, the samples from newborn infants born by vaginal delivery had a higher within-sample diversity, but the differences were not statistically significant. In previous microbiome studies using meconium samples, some studies have reported no association with delivery mode,^2,4^ but most recent studies have found differences between vaginal delivery and C-section delivery.^6–8,30,31^ Hypothetically, if the delivery mode alters the meconium microbiome but not the bEV contents of meconium, it might be possible that the bEVs may have been transferred from mother to child in utero. However, the limited sample size of this pilot study does not allow for this conclusion based on the present data.

Earlier, antibiotic treatment has been suggested as a modifying factor in meconium microbiome,^30^ as well as gut^32^ and oral^33^ microbiome of infants. Vesicle studies, on the other hand, have found that bEVs have protective effects on bacteria in stressful environments. Biofilm production and transporting capabilities of bEVs, which help with nutrition acquisition, signaling, and carrying of resistant molecules such as β-lactams and various enzymes, have been shown to increase the survival of gram-negative bacteria under antibiotic treatments, and vesicle production seems to increase as a stress response in bacteria.^34^ In our study, we compared vaginal delivery samples based on the usage of intrapartum antibiotics before or during birth. We found no significant differences in diversity or taxonomy between these samples, although intrapartum antibiotic vaginal delivery samples seemed to have a slightly higher alpha diversity than the other sample types.

The presence of bacteria-derived EVs in the newborn microbiome is a novel finding. The role of other EVs in pregnancy, however, has been explored. These previously characterized vesicles are released by many cell types, including maternal and fetal membrane cells and the placenta, and have a role of constant signaling throughout the pregnancy.^35^ Placenta-derived EVs, particularly exosomes, are known cargo transporters between immune cells to suppress the immune system to protect the development of the fetus in the womb and at the same time activate the immune system to protect the mother from infections.^36,37^ Furthermore, placenta-derived EVs may possibly act as markers for disorders during pregnancy, such as pre-eclampsia.^38^ EVs have also been found to carry inflammatory cargo that contributes to spontaneous preterm birth.^39^ Therefore, bacteria-derived EVs and their possible effects on pregnancy outcomes is an interesting topic that merits future exploration.

The strength of the study was that it was a hypothesis-driven work based on our earlier observation of EV-sized particles by electron microscopy in the first-pass meconium. We used methods that had been successfully tested in earlier meconium microbiome studies. We used first-pass meconium samples collected within 24 h of birth. The collection time in meconium microbiome studies is critical, as the neonate starts being exposed to environmental bacteria immediately upon birth.^2^ One of the limitations of this study is the collection method of meconium. The skin commensal findings indicate that the meconium samples have likely been exposed to environmental bacteria during sample collection. Another limitation is the sequencing of only one region of the 16S gene. A known issue with 16S rRNA gene sequencing is the bias for and against some taxa based on the primer choice.^40^ In addition, we did not perform copy number correction methods for our taxonomy analysis in this study. While performing the copy number correction might have made our data analysis more accurate, the methodology in 16S rRNA data is still controversial.^41^ Finally, we did not have sufficient material for bacterial DNA extraction from the same samples to allow for direct comparisons between the samples’ bacterial DNA and bEV content.

In conclusion, we show that the meconium of newborn infants contains bacterial EVs. This is a novel finding, as the presence of EVs in the meconium microbiome has, to our knowledge, not been studied before. The source of bacterial EVs may most likely be the first live whole-cell bacteria colonizing the newborn’s gut or hypothetically maternal transfer in utero, because bEV may be more able to cross biological barriers than whole-cell bacteria.

## Supplementary information

Supplementary material

## Data Availability

The raw sequences were submitted to Genbank under the bioproject accession number PRJNA816091.
