# Supplementary material for: Bacterial extracellular vesicles in the microbiome of first-pass meconium in newborn infants
Source: Pediatr Res. 2022 Aug 9;93(4):887–96. doi: 10.1038/s41390-022-02242-1 (PMC10033452; doi:10.1038/s41390-022-02242-1)
Supplement: Supplementary file 1 — Supplementary material [file 41390_2022_2242_MOESM1_ESM.pdf]

Supplementary material

**Taxa identified in EVs extracted from meconium, phylum level (%)**		**Taxa identified in EVs extracted from meconium, genus level (%)**	
Firmicutes	62	*Streptococcus*	21
Actinobacteriota	18	*Staphylococcus*	17
Proteobacteria	10	*Anaerococcus*	12
Bacteroidota	7.3	*Corynebacterium*	10
Verrucomicrobiota	0.40	*Finegoldia*	5.6
Patescibacteria	0.35	*Cutibacterium*	4.1
Acidobacteriota	0.31	*Paracoccus*	2.6
Deinococcota	0.26	*Micrococcus*	2.4
Bdellovibrionota	0.19	*Prevotella*	1.5
unknown phylum	0.17	*Escherichia-Shigella*	1.4
Other	0.53	Other	22

Supplementary information 1. The percentage of bacterial features from meconium extracellular vesicles.

EV content of meconium samples of infants born by vaginal delivery (VD)			
**Phylum (%)**		**Genus (%)**	
Firmicutes	59	*Streptococcus*	20
Actinobacteriota	20	*Staphylococcus*	18
Proteobacteria	12	*Corynebacterium*	12
Bacteroidota	7.5	*Anaerococcus*	12
Deinococcota	0.22	*Finegoldia*	5.7
Patescibacteria	0.19	*Paracoccus*	5.4
Acidobacteriota	0.17	*Cutibacterium*	4.2
Myxococcota	0.064	*Micrococcus*	2.8
unknown phylum	0.035	*Prevotella*	2.1
Desulfobacterota	0.035	*Escherichia-Shigella*	1.9
Other	0.074	Other	16
**EV content of meconium samples of infants born by vaginal delivery and exposed to intrapartum antibiotics (VD+AB)**			
**Phylum (%)**		**Genus (%)**	
Firmicutes	57	*Staphylococcus*	18
Actinobacteriota	19	*Streptococcus*	15
Proteobacteria	12	*Anaerococcus*	11
Bacteroidota	6.9	*Corynebacterium*	11
Verrucomicrobiota	1.1	*Finegoldia*	5.9
Patescibacteria	0.78	*Cutibacterium*	4.9
Bdellovibrionota	0.52	*Micrococcus*	2.7
Acidobacteriota	0.50	*Chryseobacterium*	1.9
Deinococcota	0.36	*Escherichia-Shigella*	1.9
candidatus Eremiobacterota	0.36	*Enhydrobacter*	1.2
Other	1.078	Other	27
**EV content of meconium samples of infants born by C-section and exposed to intrapartum antibiotics**			
**Phylum (%)**		**Genus (%)**	
Firmicutes	72	*Streptococcus*	31
Actinobacteriota	14	*Staphylococcus*	15
Bacteroidota	7.4	*Anaerococcus*	14
Proteobacteria	5.2	*Corynebacterium*	8.1
unknown phylum	0.28	*Finegoldia*	5.3
Acidobacteriota	0.24	*Cutibacterium*	2.9
Deinococcota	0.16	*Leuconostoc*	2.8
Desulfobacterota	0.073	*Flavobacterium*	2.5
Chloroflexi	0.017	*Lactobacillus*	1.9
Campilobacterota	0.012	*Micrococcus*	1.6
Other	0.004	Other	16

Supplementary information 2. The features sequenced from meconium extracellular vesicles with the highest frequencies on the phylum and genus levels, separated by the delivery mode and the involvement of intrapartum antibiotics.

The features sequenced from meconium extracellular vesicles with the highest frequencies on the phylum and genus levels, separated by the delivery mode and the involvement of intrapartum antibiotics.
